# Diagnostic dilemma between angular and interstitial ectopic pregnancy: 3D ultrasound features

**DOI:** 10.1007/s40477-022-00668-1

**Published:** 2022-04-21

**Authors:** Y. G. Durand, R. Capoccia-Brugger, Y. Vial, V. Balaya

**Affiliations:** 1 Obstetrics and Gynecology Department, University Hospital of Lausanne, Lausanne, Switzerland; 2 Obstetrics and Gynecology Department, Neuchâtel Hospital, Neuchâtel, Switzerland; 3 University of Lausanne, Lausanne, Switzerland; 4 Service de Gynécologie-Obstétrique et Médecine de la Reproduction, Hôpital FOCH, 40 Rue Worth, 92150 Suresnes, France

**Keywords:** Angular pregnancy, Interstitial pregnancy, 3D ultrasound, Pelvic ultrasound

## Abstract

Angular pregnancy is a rare entity which is commonly confused with interstitial or cornual pregnancies. A lack of consensus about the specific ultrasound features of these 3 entities leads to inappropriate interchange between them among the literature. An angular pregnancy should be considered as a potentially viable intra-uterine eccentric pregnancy as it might be carried to term and result in a live-born baby whereas interstitial or cornual pregnancies should be considered as ectopic pregnancies which should be interrupted. We report here two cases of women at 8 weeks of pregnancy with an angular pregnancy diagnosed by vaginal 2D and 3D ultrasound and discuss about specific ultrasound features and alternative imaging modalities to distinguish it from interstitial and cornual pregnancies.

**Supplementary Information:**

The online version contains supplementary material available at 10.1007/s40477-022-00668-1.

## Introduction

Angular pregnancy is a rare entity which was initially described in 1898 by Kelly as implantation of the embryo in the endometrial cavity at the superior and lateral angle of the uterine cavity and medially to the utero-tubal junction [1]. Due to its location close to the utero-tubal junction, angular pregnancy is commonly confused with interstitial or cornual pregnancies.

Ectopic pregnancies correspond to pregnancies which are located outside of the endometrial cavity but inside the uterus such as interstitial, caesarean scar or cervical pregnancies. Interstitial pregnancies represent 2–4% of ectopic pregnancies [2, 3]. They are implanted at the origin of the proximal fallopian tube segment in the myometrium and laterally to the round ligament.

“Cornual” pregnancy designs an intrauterine implantation in an abnormal unicornuate, bicornuate, or septate uterus [4] whereas a trophoblastic peripherical implantation in a normal uterus may rather correspond to an “interstitial” or “angular” pregnancy.

A lack of consensus about the specific ultrasound features of these 3 entities leads to inappropriate interchange between them among the literature. Nonetheless, the recent 2020 ESHRE recommendation aims to standardize the terminology to describe embryo site implantation [5, 6].

In this setting, the clinical challenge is to avoid managing an ectopic pregnancy (such as interstitial), as an intrauterine pregnancy (such as angular pregnancy) [7–10]. Indeed, interstitial pregnancies should be interrupted since it may lead to major complications such as uterine rupture, hemorrhagic shock, and death. By contrast, an angular pregnancy should be considered as a potentially viable intra-uterine eccentric pregnancy and managed expectantly after discussion with the patient [11–13] since it might be carried to term [14, 15] with a live-born baby although obstetrical outcomes remains controversial [16, 17].

We report hereby two cases of women who had an 8-week angular pregnancy diagnosed by vaginal 2D and 3D ultrasound and discuss about specific ultrasound features and alternative imaging modalities to distinguish it from interstitial and pregnancies.

## Case-report

### Case-report 1

A 38-year-old woman, G3P1, was referred to our center by her own practitioner for a second-look ultrasound for a suspicion of ectopic tubal pregnancy. In her history, she had a prior cesarean section eighteen years ago for an unexplained in utero fetal death at 28 weeks, and an early miscarriage in 2020. The current pregnancy was estimated at 8 weeks of gestational age according to the date of her last period. Clinically, she was paucisymptomatic, except for small vaginal bleeding for 3 days. The physical examination was normal. The blood pressure was 125/70 mmHg, the heart rate was 65 bpm and the oxygen saturation was 100%.

**Fig. 1.**
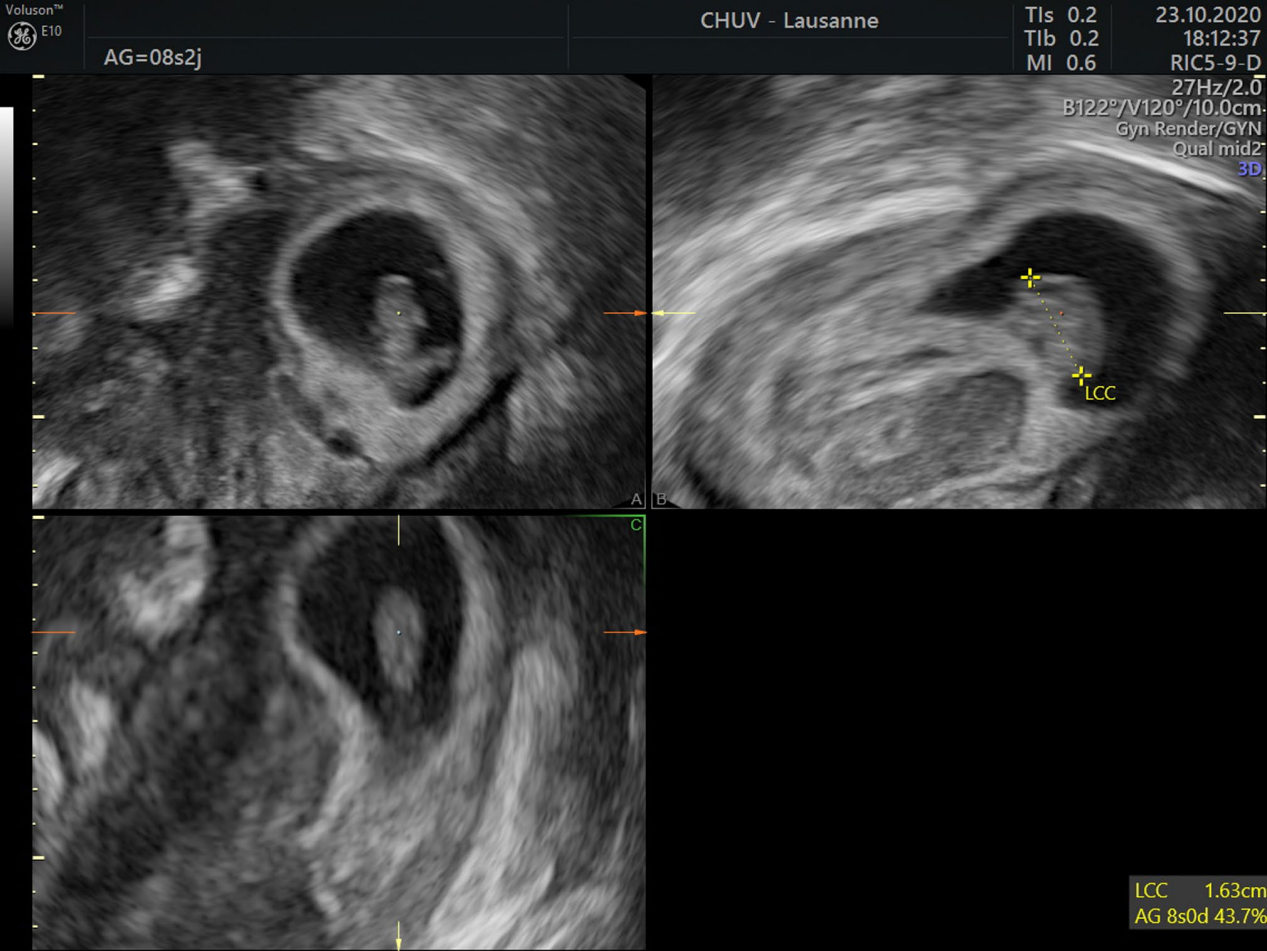
2D Ultrasound coronal view of angular pregnancy at 8wg

**Fig. 2.**
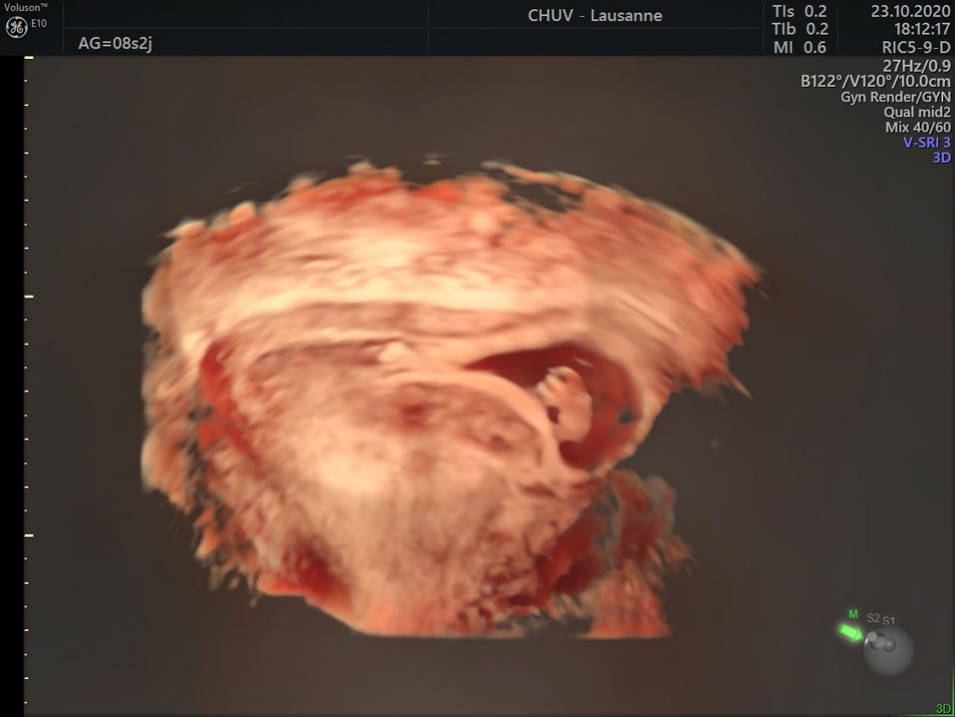
3D Ultrasound coronal view of angular pregnancy at 8wg

**Fig. 3.**
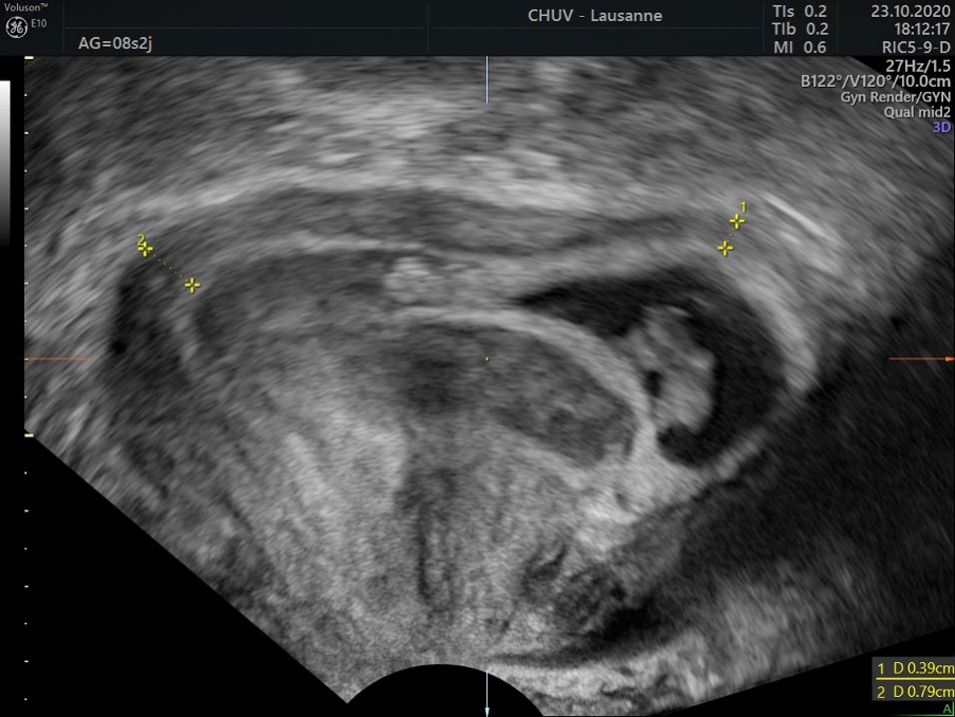
2D Measurement of myometrial thickness around angular localization at 8wg

Transvaginal pelvic sonography was performed and did not induce any pelvic pain. The transvaginal 2D revealed an anteverted uterus measuring 110 × 63 × 72 mm, with a 16 × 17 mm anterior intra-mural myoma, and no pelvic fluid in the Douglas’s pouch. There was an evolutive 8w0d-pregnancy with a crown rump length embryo of 16.3 mm and a normal heart activity of 135 bpm. The pregnancy was localized in the uterine upper left angle of the uterus and was fully surrounded by endometrium without any interstitial line sign. Around the gestational sac, a subchorionic hematoma of 25 × 22 × 20 mm was present (Fig. 1 and Supplementary material 1). Considering that the localization remained unclear, a 3D vaginal ultrasound was carried out (Fig. 2 and Supplementary material 2). In the left upper angle of the uterus, the gestational sac was fully surrounded by endometrium, and the myometrial thickness was 3.9 mm (Fig. 3). At the lower part of the gestational sac, the subchorionic hematoma was confirmed. After discussion with the patient about potential evolution and complications, she chose to keep the pregnancy and an expectant strategy was decided. Finally, a miscarriage occurred one week later without any complications.

### Case-report 2

The second case was a 37-year-old woman, G2P1. Her ultrasound report was sent to our center for a second look due to peripherical pregnancy localization. In her personal medical history, she had a prior emergency cesarean delivery at 38 weeks for a non-progression of presentation 6 years ago, complicated by a postpartum endometritis. She also had a hysteroscopy two years ago with multiple synechia resection. According to her last period the current pregnancy was estimated at 8 weeks of gestational age. She was clinically asymptomatic; with a blood pressure of 110/60 mmHg, a heart rate of 70 bpm and an oxygen saturation of 100%.

**Fig. 4.**
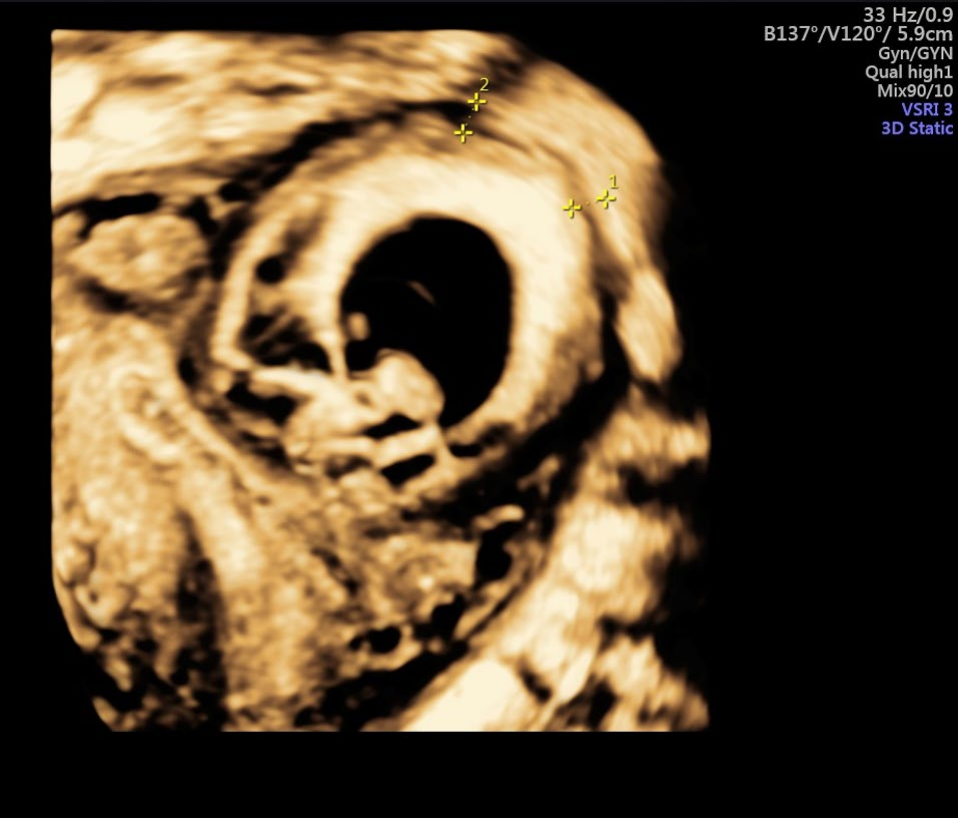
3D Measurement of myometrial thickness around angular localization at 8wg

The first 2D and 3D vaginal ultrasound examination did not induce any pain and revealed an 84 × 53 × 75 mm anteverted uterus, no pelvic fluid in the Douglas’s pouch. There was an evolutive 8w3d pregnancy with a crown rump length of 18 mm and a normal heart activity of 160 bpm. The pregnancy was localized in the uterine upper left angle and was fully surrounded by endometrium. The myometrial thickness ranged from 4.0 to 5.7 mm (Fig. 4). After discussion about potential complications the patient chose to keep the pregnancy. Pregnancy viability was assessed each week and the gestational sac localization gradually moved toward the endometrial cavity. Finally, the pregnancy was normally centered in the cavity at 13w0d whereas only the placenta remained fundal and lateralized in the upper left angle. At 37w0d, an emergency cesarean section was done for a suspicion of uterine rupture because of brutal abdominal pain and moderate hemoperitoneum of 200 ml revealed by abdominal tomodensitometry. She gave birth to a 3000 g newborn with a 7–9-10 Apgar score and a good evolution.

During the surgery, no uterine rupture was found but uterine serosa was millimetric on the upper left angle regarding to the placenta position. The surgery was complicated by a massive hemorrhage of 3000 mL, which required successively oxytocin, sulproston, and uterine artery embolization. The patient received 6 red cells units and evolved favorably. At final pathologic examination, a placenta accreta was diagnosed.

## Discussion

We reported here two cases of angular pregnancy which were diagnosed by 3D transvaginal ultrasound. This diagnostic is often confused with interstitial pregnancy and this misdiagnosis may lead to unjustified pregnancy termination. Ultrasound features in angular pregnancy are heterogeneous among the literature and most recent cases are summarized in Table 1. One of the main pitfalls is the absence of consensual diagnostic criteria.

**Table 1:** Review of literature of angular pregnancy case reports including ultrasound features

Author	Year	Number of cases	Gestational age (weeks and day)	Interstitial line sign	Myometrial thickness < 10 mm	Gestational sac fully surrounded by endometrium
Durand et al	2021	2	8w	No	Yes (3.9 mm)	Yes
			8w	No	Yes (4.2 mm)	Yes
Laus et al. [10]	2019	1	5w6d	–	Yes (6 mm)	–
Marfori et al. [15]	2018	1	6w	No	Yes (5–8 mm)	–
Cordeiro et al. [9]	2018	1	–	–	–	No (partially)
Alanbay et al. [13]	2016	1	6w	–	–	Yes
Kambhampati [8]	2012	1	6w4d	–	Yes (5.5 mm)	–
Kwon et al. [12]	2011	2	7w	–	Yes (2 mm)	Yes
			2nd trimester	–	–	–
Mayer et al. [14]	2011	1	–	–	–	–
Adam et al. [11]	2010	1	5w	–	Yes (7 mm)	––
Tarim et al. [7]	2004	1	7w	–	–	–

To distinguish angular from interstitial pregnancy, Janson and Elliott added a surgical description criteria during laparascopy [16]. In their meta-analysis of 39 cases, angular pregnancy was more likely to induce a lateral uterine enlargement which displaced the round ligament reflection upward and outward whereas interstitial tubal pregnancy was more likely located laterally to the round ligament. They suggested the following clinicosurgical criteria to define angular pregnancy: (1) clinical presentation with painful asymmetric enlargement of the uterus, (2) directly observed (i.e., surgical) lateral distension of the uterus with displacement of the round ligament laterally, (3) retention of the placenta in the uterine angle. However, due to the improvement of ultrasound and other imaging modalities, laparoscopy is not considered anymore as a first-line tool for the diagnosis of ectopic and eccentric pregnancy, including angular pregnancy.

Trimor-Tritsch et al. proposed 3 ultrasound criteria to diagnose interstitial pregnancies: (1) An empty uterine cavity, (2) A chorionic sac seen separately (> 1 cm) from the lateral edge of the uterine cavity, (3) A thin myometrial layer (< 5 mm) surrounding the chorionic sac. The combination of these 3 signs provided a specificity of 88–93% and a poor sensitivity of 40% [18]. In addition, Ackerman et al. (1993) defined the interstitial line sign as an echogenic line in the upper lateral region of the uterus bordering the gestational sac and might correspond to the interstitial portion of the fallopian tube [19]. They reported a 98% specificity, and an 80% sensitivity for the diagnosis of interstitial pregnancy, although only 12 patients were analyzed.

In a review of the literature, Lewiss et al. assessed the sonographic measurement of the endomyometrial mantle as a criterion for diagnosing an abnormal implantation location and concluded to a near-uniform acceptance of less than 5 mm as being highly suspicious for an interstitial pregnancy [2]. By contrast, Bollig and Schust considered that a cut-off < 10 mm as part of an angular pregnancy criteria [17]. In our cases, the smallest myometrial thickness were 3.9 mm and 4.2 mm respectively. Comparing our findings with previous published case-reports, the myometrial thickness cut-off was not precised [7, 9, 12–14] or appeared to be heterogeneous with a thickness between 5 and 10 mm [8, 10, 11, 15]. We support the idea that clear standardized recommendations are needed about the cut-off and the myometrial thickness landmarks measurement. As highlighted by our second case, the presence of placental accretism is a frequent event in angular pregnancy due to the reduced thickness of the endometrial decidua in this area.

Recently, the ESHRE society guidelines [6] suggested the following criteria for the interstitial pregnancy diagnosis: (1) a thin intramural/interstitial segment of Fallopian tube adjoining the medial aspect of the gestational sac and the lateral aspect of the uterine cavity” (interstitial line sign). (2) The gestational sac must be at least partially enveloped by the myometrium. These guidelines also individualized a subtype as “partial” interstitial which designs a partial protrusion of the gestational sac from the tubal ostium to the uterine cavity. Performing a sonohysterography by using saline infusion may help to over cross these limitations [20].

In a prospective cohort of 42 patients with angular pregnancy, Bollig and Schust suggested the following criteria: (1) Non anomalous uterus, (2) Implantation of the embryo in the lateral angle of the uterus, (3) < 10 mm of myometrial thickness from the gestational sac to the outer border of the uterus, (4) Completely circumferential endometrium surrounding the gestational sac, (5) Lack of the interstitial line sign [17]. Main differences between interstitial and angular pregnancy ultrasonographic features are summarized in Table 2. In the first case, all these signs were present. The 3D ultrasound was of paramount interest for acquiring an accurate coronal view of the uterus fundus [21] since this imaging modality enhanced to confirm that endometrium was fully surrounded the pregnancy and thus considered as intrauterine. However, ultrasound might be limited by several factors such as interference with intestinal gas, obesity, or operator experience.

**Table 2:** Main ultrasonographic differences between angular and interstitial pregnancy

	Angular	Interstitial
Classification	Eccentric	Ectopic
Position to the round ligament	Medial	Lateral
Position to the endometrial cavity	Inside	Outside
Position to the utero-tubal junction	Medial	Lateral
Myometrial mantle thickness	> 5 mm	≤ 5 mm
Endometrium-myometrium junctional zone	Presence of gestational sac	Intact
Distance between lateral uterine border and gestational sac	< 1 cm	> 1 cm
Surrounding structure	Endometrium	Myometrium
Interstitial line sign	No	Yes

In case of unclear situation after performing a vaginal ultrasound, magnetic resonance imaging (MRI) appears to be a suitable alternative as second line exam [22, 23]. MRI presents some advantages: no patient premedication, no ionizing radiation, the possibility of multiplaning imaging, and a really good soft tissue contrast [24]. Nonetheless, gadolinium injection should be avoided since it crosses the placenta. In T2-weighted ponderation sequence, interstitial pregnancy appears as a gestational sac in the uterine angle, with an intact junctional zone between the gestational sac and the endometrial cavity [22].

In our two cases, the lack of the interstitial line sign and the fully surrounded endometrium sign were present. Although these both signs are rarely described in most of recent case-reports (Table 1), they enhanced us to diagnose the angular localization of those pregnancies. These case-reports deserved to underline that these both signs may be more discriminant for angular pregnancy diagnosis than isolated myometrial thickness measurement. Nevertheless, larger prospective cohorts are needed to clarify ultrasonographic features of angular pregnancy.

Despite all these descriptions, diagnosis of angular pregnancy may remain difficult and repeated exams by experienced ultrasonographist and close follow up should be required before considering exploratory laparoscopy.

## Conclusion

Angular pregnancy is uncommon, and the eventuality of such unusual localization should be remembered to avoid confusing with cornual and interstitial topography. Those cases highlight the role of the 3D ultrasonography to help for diagnosing angular pregnancy which may be considered as a viable intra-endometrial eccentric pregnancy and may be managed to term.

## Supplementary Information

Below is the link to the electronic supplementary material.

Supplementary file1 (MP4 5148 KB)

Supplementary file2 (MP4 1146 KB)
